# Prevalence and Predictors of Food Insecurity among Older People in Canada

**DOI:** 10.3390/ijerph15112511

**Published:** 2018-11-09

**Authors:** Janette Leroux, Kathryn Morrison, Mark Rosenberg

**Affiliations:** 1 School of Kinesiology and Health Studies, Queen’s University, Kingston, ON K7L 3N6, Canada; 2 Department of Epidemiology, Biostatistics and Occupational Health, McGill University, Montreal, QC H3A 0B9, Canada; kathryn@precision-analytics.ca; 3 Department of Geography and Planning, Queen’s University, Kingston, ON K7L 3N6, Canada; mark.rosenberg@queensu.ca

**Keywords:** food insecurity, aging, older people, Canada

## Abstract

*Background*: Food insecurity research has been mainly examined among young people. The root causes of food insecurity are closely linked to poverty, and social policies and income supplements, including public and private pensions, have been shown to sharply curb food insecurity into later life. However, social, economic, and political trends that are closely connected to social and health inequalities threaten to undermine the conditions that have limited food insecurity among older people until now. Exploring the prevalence and predictors of food insecurity among older people across Canada has important implications for domestic policies concerning health, healthcare, and social welfare. *Methods*: Data come from the Canadian Community Health Survey 2012 Annual Component (*n* = 14,890). Descriptive statistics and a generalized linear model approach were used to determine prevalence and estimate the associations between food insecurity—as measured by the Household Food Security Survey Module—and social, demographic, geographic, and economic factors. Results: Approximately 2.4% of older Canadians are estimated to be moderately or severely food insecure. Income was by far the strongest predictor of food insecurity (total household income <$20,000 compared to >$60,000, OR: 46.146, 95% CI: 12.523–170.041, *p* < 0.001). Younger older people, and those with a non-white racial background also had significantly greater odds of food insecurity (ages 75+ compared to 65–74, OR: 0.322, 95% CI: 0.212–0.419, *p* < 0.001; and OR: 2.429, 95% CI: 1.438–4.102, *p* < 0.001, respectively). Sex, home ownership, marital status, and living arrangement were all found to confound the relationship between household income and food insecurity. Prevalence of food insecurity varied between provinces and territories, and odds of food insecurity were approximately five times greater for older people living in northern Canada as compared to central Canada (OR: 5.189, 95% CI: 2.329–11.562, *p* < 0.001). *Conclusion*: Disaggregating overall prevalence of food insecurity among older people demonstrates how disparities exist among sub-groups of older people. The seemingly negligible existence of food insecurity among older people has obscured the importance, practicality, and timeliness of including this age group in research on food insecurity. The current research underscores the critical importance of an income floor in preventing food insecurity among older people, and contributes a Canadian profile of the prevalence and predictors of food insecurity among older people to the broader international literature.

## 1. Introduction

The Guaranteed Income Supplement and Canada Pension Plan that was legislated in 1967 addressed a long-running trend of pervasive poverty among older citizens (aged 65 and older) in Canada and continues to be considered a major achievement for policymakers [1,2]. However, there exist challenges facing current and future Canadian older people. In Canada, the proportion of older adults to the total population is rapidly increasing [3] amidst constant threats to the social safety net [4], food prices and cost of living are ever increasing [5,6], and low-wage, part-time, and precarious employment are rampant across sectors [7,8]. Worsening conditions of poverty and inadequate savings trends among the poor and working poor are likely to lead to a new wave of older people living in poverty [9,10].

Research suggests that when people are unable to meet their most basic living needs, such as housing and heating, they make difficult tradeoff decisions with more flexible expenses, primarily food [11]. While hunger is defined as the physical pain or discomfort an individual experiences in response to inadequate intake [12], food insecurity encompasses hunger as a severe outcome, but also captures the ongoing stress associated with not knowing if you will have enough to eat, and the quantitative, qualitative, psychological and social coping and consequences of not being able to put enough food on the table [13]. Household food insecurity is defined as “the limited or uncertain availability of nutritionally adequate and safe foods or limited or uncertain ability to acquire acceptable foods in socially acceptable ways” [14]. Food insecurity is argued to be the most sensitive indicator of poverty [15], and is a persistent problem in Canada, despite strong national economic prosperity [16]. In Canada, benefits for older people have been shown to act as an income floor to buffer against food insecurity among older people, as older Canadians (aged 65 and older) are least likely to be food insecure [17]. In fact, some research has demonstrated a reversal in trends among some of the most vulnerable sub-groups of near-old (aged 55–64 years), which is directly attributable to the weakening of older adult-focused social policies and income supplements [18].

Food insecurity has been monitored in Canada at the population level since 2004, when the Household Food Security Survey Module (HFSSM) was first included in the Canadian Community Health Survey as optional content for each province [19]. Since then, food insecurity research has mostly focused on younger populations [20,21,22]. This is in part due to older people experiencing the lowest rates of food insecurity as compared to younger people. Indeed, rates of food insecurity among older people are a fraction of those in younger people, with household food insecurity affecting one in six children (under the age of 18), and approximately 12.0% of all households in Canada [23]. Similarly, research on food insecurity in other countries with strong social safety nets for older people also report lower rates of food insecurity among older people. For example, in Australia, rates of food insecurity among older people have been reported as 2.0%, 2.6%, 4.5%, and 13.0% [24,25,26,27]. Rates of food insecurity among older people in the United States are estimated to fall between 6% and 18% [22,28,29,30,31,32]. However, inconsistent measurement across surveys makes it difficult to draw conclusions from comparisons between and sometimes within countries. Additionally, in Canada, there are few studies that specifically examine the prevalence of food insecurity among older people, or the social, demographic, or economic factors that predict food insecurity among older people [33,34]. This information is important for informing the development and evaluation of health care and social welfare policies for older people [35]. Similarly, it is important to contribute a Canadian profile of food insecurity among older people to the international literature.

Our primary research objectives are to determine the prevalence of household food insecurity among older people in Canada overall, and by province. Furthermore, we intend to determine what sub-groups of older people are most vulnerable to household food insecurity, as well as the predictors of household food insecurity among older people.

## 2. Materials and Methods

The current study used a retrospective cross-sectional design using secondary data from the Canadian Community Health Survey (CCHS), 2012 Annual Component [36]. The CCHS is a cross-sectional survey that collects information related to health status, health care utilization, and health determinants for the Canadian population. CCHS data is collected from persons aged 12 and over living in private dwellings in 115 health regions of all provinces and territories [37]. The survey does not include indigenous people living on reserves (a reserve is a tract of Crown land (not privately owned) set aside for the use and benefit of an Indian band (community)), institutional residents, full-time members of the Canadian Forces, and residents of certain remote regions. Younger and older people were oversampled, and the CCHS is estimated to cover approximately 98% of the Canadian population (aged 12 and over) [37]. We followed the recommendations of Statistics Canada and applied population weights to appropriate analyses [37]. Because this study is a secondary analysis of data collected by Statistics Canada (Statistics Canada is Canada’s National Statistical Agency, and and how the Agency mitigates survey respondent risk can be found at https://www.statcan.gc.ca/eng/rdc/mitigation), and makes use of a publicly available dataset that does not contain identifiable information, Queen’s University General Research Ethics Board does not require an ethics review of the data collection methods or the analytical approach taken in this research [38].

In 2012, the Household Food Security Survey Module (HFSSM) was administered as common content to all regions. The HFSSM is a household measure, and is designed to capture self-reports of uncertain, insufficient, or inadequate food access, availability, and utilization due to limited financial resources, and the compromised eating patterns and food consumption that may result [39]. The HFSSM contains 18 questionnaire items—10 comprising the adult scale, and 8 comprising the child scale—and asks about the food security situation in a household over the past 12 months, as shown in Table 1.

Often, sometimes, and “yes” are considered positive (affirmative) responses. Based on the responses, the following three categories can be used to describe household food security situations, as defined by Health Canada: food secure, food insecure (moderate), and food insecure (severe), as shown in Table 2.

We used the adult scale of the HFSSM. The dependent variable, (adult) household food security, was defined as a dichotomous variable, food secure and food insecure. Food insecure (moderate), and food insecure (severe) were collapsed into one category, as per recent discussions in food security research in Canada [40]. The selection of covariates and their categorization was informed by other studies, as per the important characteristics of food insecure older people. Accordingly, the following potential risk factors were explored in the analysis: age, sex, racial background, self-perceived health, education, income, marital status, living arrangement, home ownership, and province of residence. Each potential predictor was categorized as follows: age (65–74, and 75 and over), sex (male, female), racial background (white, visible minority), self-perceived health (excellent, very good, good, fair, poor), education level—household (graduated post-secondary, other post-secondary, graduated secondary, less than secondary). Income was measured by total household income from all sources (>$60,000, $40,000–$59,999, $20,000–$39,999, <$20,000). The provinces included were Newfoundland (NFLD), Prince Edward Island (PEI), Nova Scotia (NS), New Brunswick (NB), Quebec, Ontario, Manitoba (MN), Saskatchewan (SK), British Columbia (BC), and Alberta (AB). Territories were combined (Yukon, Northwest Territories (NWT), and Nunavut). Alberta was arbitrarily selected as the referent group.

All statistical analyses and calculations were performed using SPSS version 24.0 [41]. The CCHS 2012 Public Use Microfile (PUMF) was obtained and included a total of 61,707 cases (unweighted) and 1381 variables. Data were restricted to people aged 65 years and older (*n* = 16,785). We explored food insecurity by the previously mentioned social, demographic, economic, health, and geographic variables. Respondents with missing data for the selected study variables were excluded (*n* = 1895). A complete case analysis was performed, and the final unweighted sample size was 14,890. 

**Figure 1:**
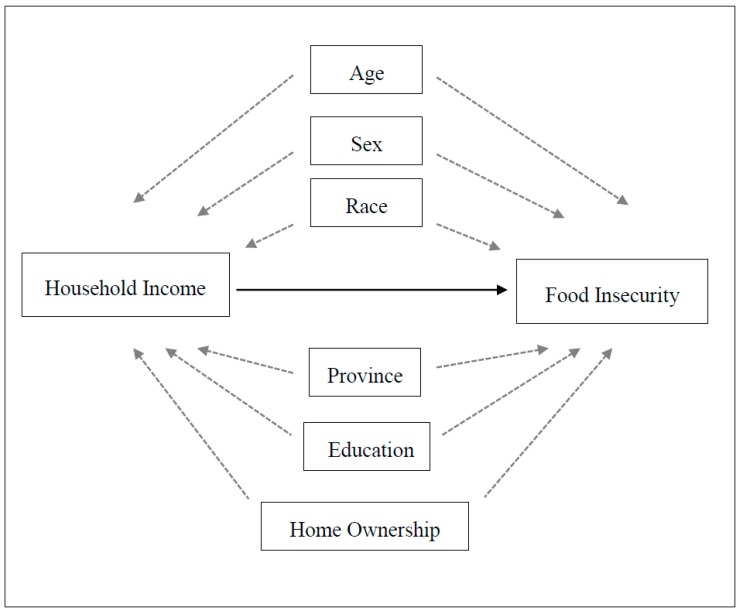
Directed acyclic graph (DAG) demonstrating the potential relationships in our assumed model, where we explore the likely confounders/predictors of the relationship between household income and food insecurity among older people.

Next, response frequency by questionnaire item was broken down for food insecure older people. Group differences between food security status and selected variables were analyzed using Chi-Square tests, in order to compare the prevalence of food insecurity among older people between levels of the independent characteristics. Prevalence ratios were calculated for each characteristic by dividing the category with the greater prevalence by the category with the lesser prevalence. We used a generalized linear model for binomial data with a logit link function. A robust variance was used in order to account for weighting of cases [42]. The statistical significance threshold was set for *p* < 0.05. Reference categories were selected based on previous literature and/or hypothesized relationships. Odds ratios, confidence intervals, and *p*-values were reported for each category of characteristic. Our base model controlled for age, sex, and race as the most likely potential confounders of the relationship between household income and food insecurity status. We then explored the impact that adding additional covariates had on the magnitude and precision of the household income parameters and looked at model fit using AIC (Akaike information criterion) [43]. Based on AIC values for each model, we were able to narrow down the number of variables to be considered, and selected the model with the smallest AIC. The final model thus represents the best overall statistical properties and parameter balance. We show a directed acyclic graph (DAG) to depict our assumptions in the relationships between potential confounders/predictors of the relationship between household income and food insecurity, as shown in Figure 1.

## 3. Results

There was a total of 16,991 older people who were included in the 2012 CCHS cycle. This number was reduced to 14,890 who had completed the HFSSM and were not missing any data for each of the selected covariates. Data analyses of the missing data cases revealed no significant differences between them and the sample used for the current study. Of the total sample, 382 were classified as food insecure. Table 3 provides a summary of study participant descriptive measures according to the selected covariates, and includes unweighted and weighted prevalence across levels of the covariates.

The overall prevalence of food insecurity was 2.4%. A description and summary of responses to each individual survey item is shown in Table 4. Of the older people who were classified as food insecure, the most common statements included “cutting the size of meals or skipping meals”, and “eating less than you felt you should because there wasn’t enough money to buy food”. About one tenth of those who were classified as food insecure also reported p28hysical symptoms of hunger (9.0%) and withholding from eating for an entire day (11.5%). Responses were also examined by age and sex, but these sub-stratifications did not reveal any notable differences.

The prevalence of food insecurity by selected characteristics is outlined in Table 5, alongside prevalence ratios and chi-square statistics. Food insecurity was most prevalent among younger-old, women, non-white, those who reported poorer self-perceived health, those who were un-married, and those who were living alone or with people other than their spouse. The most remarkable differences were discovered for home ownership, and household income. For home ownership, those who indicated that they were renting rather than owning their dwelling had a crude prevalence odds ratio of 4.2.

The magnitude of difference between prevalence of food insecurity at different levels of household income was massive. The prevalence of food insecurity was found to be 54 times greater among those with an income of less than $20,000 per year, 18.5 times greater among those with an income of between $20,000 and $40,000 per year, and 4 times greater among those with an income between $40,000 to $60,000 per year, all compared to those with an income of $60,000 and greater per year. There was also notable variability in prevalence of food insecurity between provinces and territories, where the prevalence of food insecurity ranged between 2% to 3% for most provinces, with the exception of Prince Edward Island (3.8%) and New Brunswick (4.1%). The combined category for the territories had a much higher prevalence of food insecurity at 11.2%. The prevalence of food insecurity did not follow a clear trend with respect to level of education, as highest levels of food insecurity were found to occur among those with other postsecondary—which included Trade Certificate or Diploma, College, CEGEP (College d’enseignement général et professional is a general and vocational college for students in the province of Quebec) or diploma other than trades certificates and diplomas (4.7%), and those with less than secondary education (3.4%). As the chi-square statistic was found to be significant for all factors as outlined in Table 5 in terms of crude risk, all ten factors were thus considered to be potential candidate predictors for the multivariable model selection process. A base model included the four most important predictor variables known to be associated with food insecurity among older people, as shown in Table 6. We found sex to be confounded by income, and age and race both independent predictors of food insecurity.

Marital status and living arrangement were both found to be confounded by income, losing their significance once household income was included in the model and not improving the model fit based on AIC; they were excluded from the model. Home ownership appeared to also be confounded by income, as it went from being very significant to borderline insignificant again once income was re-added to the model. Model fit improved slightly by its inclusion, and the standard errors of the other variables were not changed considerably, thus it was included in the final model.

**Figure 2:**
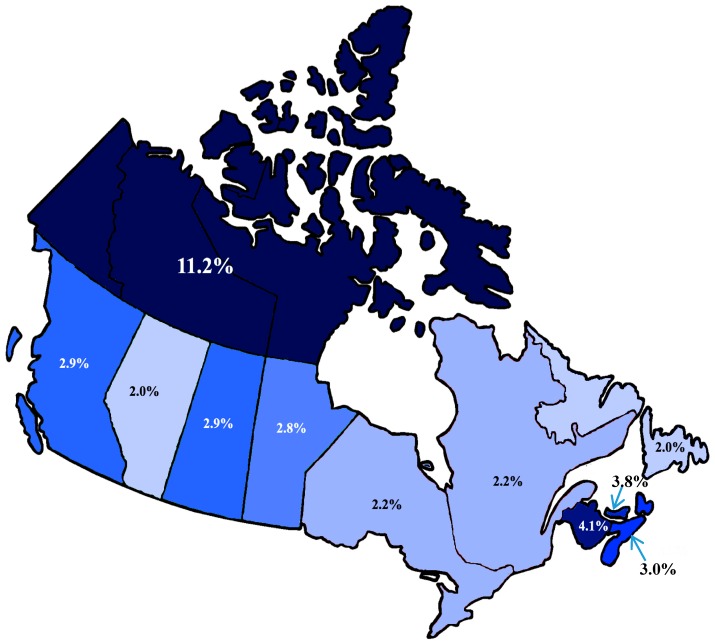
Map of Canada with prevalence of food insecurity among older people (65 years and older) by province.

Prevalence of food insecurity varied by province, as shown in Figure 2. Province of residence was only significantly influential between the referent group (Alberta) and the territories (Yukon/NWT/Nunavut); however, it slightly improved the model fit, and did not change the standard errors, thus it was included in the model. When included individually, only the Northern territories were significantly associated with increased odds of food insecurity. We grouped provinces and territories into regions: Northern, Atlantic, Central, and Western. The Northern region included Yukon, Northwest Territories, and Nunavut. The Atlantic region included Newfoundland, Prince Edward Island, Nova Scotia, and New Brunswick. The Central region included Ontario and Quebec, and the Western region included British Columbia, Alberta, Saskatchewan, and Manitoba. Thus, the final model included the following predictors: age, sex, race, household income, education, home ownership, self-perceived health, and region of residence, as shown in Table 7.

Older people, aged 75 years and older, had reduced odds of being food insecure as compared to younger older people aged 65 to 74 years (OR: 0.322, 95% CI: 0.212–0.419, *p* < 0.001). Sex was not found to be significantly associated with food insecurity in this model, despite food insecurity being 1.1 times more prevalent among women as compared to men. People who identified as being a visible minority were also found to have almost 2.5 times greater odds of being food insecure as compared to those who identified as being white (OR: 2.429, 95% CI: 1.438–4.102, *p* < 0.001). A strong relationship was discovered between food insecurity and household income in the final model.

Home ownership and self-perceived health were not found to be significantly associated with food insecurity in the final model. Education did not follow an expected trend, as people with the lowest levels of education (less than secondary) were found to have significantly lower odds of being food insecure as compared to those reported having graduated from post-secondary education (OR: 0.553, 95% CI: 0.364–0.840, *p* < 0.005), with no other levels of education being significantly associated with increased odds of food insecurity.

Even while controlling for all other variables in the model, region of residence also significantly influenced odds of food insecurity. Older people residing in Northern Canada had an almost 5.2 greater odds of experiencing food insecurity as compared to those in Central Canada. Respondents from Western and Atlantic regions were found to have similar odds of being food insecure, at 1.75 and 1.64, respectively, as compared to Central provinces.

## 4. Discussion

The objectives of the current study were to examine predictors and prevalence of food insecurity among older people in Canada, according to social, demographic, economic, and geographic factors that have been shown to be associated with food insecurity among older people in other countries. We found the prevalence of any level of food insecurity in this sample of older people in Canada to be approximately 2.6%.

Indeed, this is considerably lower than the prevalence found in other age groups in Canada [33] and is at the lowest end of prevalence of food insecurity among older people compared to food insecurity among older people in other countries [22,24]. While these comparisons are not absolute, due to different sampling procedures and measurement instruments employed, these findings suggest that older people in Canada are largely protected from food insecurity. In examining the predictors of food insecurity among older people in Canada, we can begin to make informed postulations about what renders some older people more vulnerable to food insecurity, and mechanisms through which other groups of older people are protected from food insecurity. While we found all factors to be significantly associated with food insecurity, when these factors were combined into a multivariable model, the most important predictor was household income by orders of magnitude, as shown in Table 7.

Much international literature, particularly coming out of the United States, points toward the complexity of food insecurity among older people as compared to younger adults [44,45,46]. Yet, the current findings reinforce the research that points to the paramount role of income in household food insecurity. Categories of household income were created from lower levels of income, and still the difference between odds of food insecurity among older people with a household income below $20,000 and those with household income between $20,000 and $40,000 were 3-fold, as shown in Table 7 and Figure 2. Indeed, cases of food insecurity were concentrated among the lowest levels of income, so people who reported an annual household income of $40,000 or less accounted for 93% of cases of food insecurity in this sample, as shown in Table 5.

While there were other important predictors that were uncovered in the current analyses, the relationship between income and odds of food insecurity was so profound that many of these predictors were found to confound the household income-food insecurity relationship. For example, while food insecurity was more prevalent among women than men, and significantly associated with food insecurity in bivariate analysis, as shown in Table 5, when added to the model was found to be insignificant when combined in the multivariable model with age, race, and household income, as shown in Table 6. This suggests that women may be more vulnerable to food insecurity through economically-driven mechanisms. Similarly, home ownership, marital status, and living arrangement have been found to be significantly associated with food insecurity among older people in previous studies [17,24,47], however failed to reach significance in the multivariable modeling process, when combined with household income, as shown in Table 7. While marital status and living arrangement can play an important role in the combining of resources and the social aspects of procuring and preparing food [48], and were found to have a significantly protective effect against food insecurity in bivariate analysis, as shown in Table 5, this influence diminished when examined alongside household income in the multivariable model, as shown in Table 7. This suggests that many of the protective effects of these variables act through economic means, as marital status, living arrangement, and home ownership variables were found to confound the relationship between household income and food insecurity. We anticipated education being an important differentiator for odds of being food insecure, as education is foundational to occupational and economic opportunities and asset accumulation across the lifespan [49]. We tested both individual education as well as highest level of educational attainment in the household (household education). However, we did not find a clear relationship between level of education and food insecurity, in either the bivariate analysis or multivariable analysis (household education variable shown in Table 5 and Table 7).

We did uncover a strong relationship between older people who identified as being non-white and their odds of food insecurity, as compared to older people that identified as being white, as shown in Table 7. This relationship was strengthened from the base model to the final model, suggesting that race/cultural origin impacts odds of food insecurity beyond household income. Some of these mechanisms may originate through macro-to-micro forms of systemic and individual racism and social marginalization, and exclusion from occupational opportunities [50]. Specific to food insecurity among older people, previous research demonstrates how food insecurity is experienced differently among different cultural groups, related to differences in social network structures and mobilization of social capital as it pertains to food [51,52]. While not examined in the current study but of great relevance is the pervasiveness of food insecurity among First Nations, Aboriginal, and Inuit populations in Canada [53,54]. Historical and ongoing systemic racism towards Indigenous peoples of Canada are connected to economic and environmental injustices, and resultant social and health inequalities. Important food insecurity research is being undertaken by and alongside Indigenous researchers, and specifically considers food issues using Indigenous methods and frameworks [55,56,57].

Contrary to international literature suggesting increasing vulnerability with increasing age among older people [24,58], the oldest people in the current sample had lowest odds of food insecurity as compared to respondents in the younger age category (65–74 years), as shown in Table 7. This vulnerability has been attributed to physical and functional limitations [59,60,61], and has potentially more costly health expenses due to higher concentration of chronic disease in oldest cohorts [62,63]. However, Canada provides Medicare to its citizens and permanent residents, which is a national publicly funded, single-payer health care system that provides universal health care coverage. This means that while older people, and older Canadians, are more likely to have increased healthcare expenditures, those expenditures are considerably less among older Canadians as compared to countries, such as the United States, that do not offer universal healthcare coverage.

Older adult-focused benefits are believed to be protective against food insecurity in older people, because they provide a modest but stable income for people starting at the age of 65 [17]. The costs and barriers to food security associated with aging may be buffered by benefits for older people. For example, age may be reinterpreted as length of time since qualifying for concessions and benefits for older people. Conversely, this finding may also be explained by the “healthy survivor effect”, or “depletion of susceptibles”, whereby those people who survive into older ages are more likely to have been healthier and have had more financial security at younger ages [64]. Drawing on the established literature around aging, poverty, and the social determinants of health, people with lower incomes tend to have poorer health outcomes as compared to people with higher incomes [65]. In this way, older people experiencing food insecurity may be less likely live as long as older people who are food secure, and thus be less likely to be represented in older age categories. The healthy survivor effect is a common thorny methodological issue for epidemiological studies on health and aging, and results tend to be interpreted alongside this specific bias. Future studies on food insecurity and aging would benefit from longitudinal data to better assess the impacts of food insecurity across the life course on life expectancy.

The prevalence of fair or poor self-rated health was 2.6 times more prevalent as compared to good or excellent self-rated health among food insecure people, as shown in Table 5. Self-perceived health did not remain significant when added to the final model; however, was included in the model as food insecurity has been demonstrably linked with poorer health outcomes and increased healthcare utilization [66].

When provinces were collapsed into regions, an interesting relationship surfaced, whereby older people from Northern territories had 5 times greater odds of being food insecure, and those from Western and Atlantic provinces had a 1.7 and 1.6 times greater odds, respectively, compared to Central provinces, as shown in Table 7. This is particularly relevant in Canada, where benefits vary by province, are adjusted to inflation, and are guaranteed for every older person the month they turn 65 years of age until death [67]. Canada also boasts universal healthcare (administered provincially), and benefits for older people that include provincial drug policies for older people, and many concessions for people aged 65 and over. However, the administration of healthcare, Pharmacare, and institutional long-term care are provincial, and accordingly has led to vastly different coverage between provinces [68], which would have differential impact on older people, with fixed incomes, who are known to spend disproportionate amounts of household budgets on healthcare expenses [69]. Regional variance in food insecurity among older people might also be superficially explained by differences in age-specific and historic costs of living, working conditions, and access to social protective measures. However, such geographically-based inequalities in household food security among older people is an important element of this issue to be examined more closely in future research.

Another issue that deserves further attention is whether age and gender act as confounders or effect modifiers. In another study, Leroux [70] focused on age and gender effects in food security. While the results are generally consistent with those found in this study, gender had no effect on food security in the final adjusted model in Leroux [70], while there was evidence that age acted as an effect modifier for the relationship between household income and food insecurity.

There are a number of implications and future research directions that arise out of the current study. To begin, this research begins to fill a gap in the literature in terms of describing food insecurity among older people in Canada in general. In Canada, there is important literature that has examined food insecurity among particularly vulnerable sub-groups of older people (lone, lowest-income), and has demonstrated that old-age benefits act as an income floor to buffer against food insecurity [17], as well as provided evidence of the reversal of high rates of food insecurity among vulnerable near-older people as they qualify for old-age benefits [18]. Despite the potential for valuable insights into income-based solutions to household food insecurity, among older as well as younger populations, food insecurity among older people in Canada remains largely yet to be explored. While the problem of food insecurity among older people has been described by public health nutrition researchers in other countries, less work has been done to explore food insecurity in this demographic in Canada. The current research connects to economic and social political research that considers the important ways that an income floor prevents poverty and material deprivation, working against strong aging factors. Not everyone has equally benefitted from older-adult focused social policies, and research which seeks to more closely examine residual rates of poverty-related problems in the aged, such as food insecurity, can serve as a window into the complexity of health and aging from a social determinants of health lens [71,72]. In terms of public health implications for the current research in the context of related literature, it will be important for public health practice to reflect the evidence that household income is powerfully predictive of food insecurity, and to avoid the pitfalls of food-based solutions to food insecurity (which have been shown to be ineffective [73,74,75]). For example, in this regard public health practice might include; advocacy for social policies that act upon inadequate income or social determinants of health more broadly; and research and health communication, which complicates food insecurity as a symptom of market failure versus individualized behavioral/nutritional failure. 

The current study suffers from the limitations of any cross-sectional dataset, whereby causal inferences should be made with caution. Future studies would benefit from longitudinal datasets exploring the dynamism of food insecurity rates over time [18,76], alongside more exhaustive and broader economic indicators [19,77], as well as the individual histories of health, socioeconomic status, and food insecurity. As well, this study made use of the publicly available dataset of the CCHS, which comes with some limitations. For example, the PUMF offers a smaller sample size, and therefore more limited power for statistical analyses. Similarly, the creation of the population survey weights are designed to be used with the 2-year Master dataset file, and are thus less accurate when applied to the single year PUMF data.

We did not include marginal food insecurity in our definition of food insecurity. While this is consistent with the definition of food insecurity put out by Health Canada, it arguably underestimates the problem. There is research to suggest that people who indicate marginal food insecurity (responding positively to one item on the HFSSM) can also have serious psychological and health implications linked to their food insecurity status, and that the shoulder group of people who experience marginal food insecurity are more similar to those who experience moderate food insecurity than those who are food secure [78].

Additionally, we included broad predictors of geography. Future studies that look more closely at regional and community-level variations of prevalence and predictors of food insecurity and older people would be able to postulate important relationships between local social, physical environments (i.e., built environment and food environment) and older people [79,80]. The relatively low prevalence of food insecurity in this sample prevented us from exploring more nuanced interactions between a range of factors due to the limited sample power.

## 5. Conclusions

Disaggregating overall prevalence of food insecurity among older people demonstrates how disparities exist among sub-groups of older people. The seemingly negligible existence of food insecurity among older people has obscured the importance, practicality, and timeliness of including this age group in research on food insecurity. We were able to demonstrate the heterogeneity of risk among older people. While not all predictors were included in the final model, earlier analyses demonstrated a host of demographic, social, economic, and geographic factors that differentially impact older peoples’ odds for food insecurity. Most importantly, household income is most strongly predictive of food insecurity among older people. Overall, these study findings contribute insight into the predictors of food insecurity among particularly vulnerable social groups, which will help to inform the implementation of necessary policies, programs, and other initiatives. The current research contributes a Canadian profile of the prevalence and predictors of food insecurity among older people to this broader international literature.

## Figures and Tables

**Table 1:** Questionnaire items and potential responses for the Household Food Security Survey Module (HFSSM)—Adult Scale.

Core Household Food Security Survey Module Questions and Response Categories (Adult Scale)		How Often in the Past 12 Months
1.	Worried whether food would run out before getting money to buy more (in last 12 months)	Often/Sometimes/Never
2.	The food bought didn’t last, and there was not enough money to get more (in last 12 months)	Often/Sometimes/Never
3.	Could not afford to eat balanced meals (in last 12 months)	Often/Sometimes/Never
*If respondents responded often or sometimes to any of the above questions, they were subsequently asked the following questions:*		
4.	In past 12 months, did you or other adults in the household ever cut the size of your meals or skip meals	Yes/No
5.	In past 12 months, how often did you cut size of meals or skip meals	Almost Every Month/Some Months/Only 1 or 2 Months
6.	In the past 12 months, did you ever eat less than you felt you should because there wasn’t enough money to buy food	Yes/No
7.	In past 12 months, were you ever hungry because you didn’t eat because you couldn’t afford enough food	Often/Sometimes/Never
8.	In past 12 months, did you lose weight because you didn’t have enough money for food	Yes/No
9.	In past 12 months, did you or other adults in your household ever not eat for a whole day because you did not have enough money	Yes/No
10.	In past 12 months, how often did you or other adults not eat for a whole day	Almost Every Month/Some Months/Only 1 or 2 Months

**Table 2:** Health Canada categories of food security status. For the current study, both categories of food insecurity were collapsed into one.

Category Labels	10-Item Adult Food Security Scale
Food Secure	0–1 affirmed responses indicating difficulty with income-related food access
Food insecure, moderate	2–5 affirmed responses indicating compromise in quality and/or quantity of food consumed
Food insecure, severe	≥6 affirmed responses indicating reduced food intake and disrupted eating patterns

**Table 3:** Study participant descriptive measures (aged 65+ and responded to Household Food Security Module). *n* = 14,890.

Variables	*n*	Weighted (%)
Sex		
Male	6156	45.9
Female	8734	54.1
Age		
65–74	8301	60.0
75+	6589	40.0
Racial Background		
White	14,009	90.7
Non-white	881	9.3
Self-Rated Health		
Excellent/Good	6593	45.5
Fair/Poor	8297	54.5
Education (Household)		
Less than secondary	3815	20.7
Graduated secondary	2277	14.3
Other Post-secondary	470	3.5
Graduated Post-secondary	8328	61.5
Marital Status		
Married, Partner	7859	65.6
Not partnered	7031	34.4
Living Arrangement		
Living with Spouse	7334	60.1
Living with Others	590	8.0
Living Alone	6966	31.9
Home Ownership		
Owned	11,435	77.0
Rented	3455	23.0
Total Income (Household)		
<$20,000	2564	13.2
$20,000–$39,900	5481	34.2
$40,000–$59,900	3322	24.4
$60,000+	3433	28.2
Province		
Newfoundland	403	1.7
PEI	210	0.5
Nova Scotia	607	3.2
New Brunswick	614	2.6
Quebec	2601	24.7
Ontario	5417	37.9
Manitoba	868	3.5
Saskatchewan	939	3.1
Alberta	1123	8.4
British Columbia	1983	14.3
Yukon/NWT/Nunavut	125	0.1
Food Insecurity		
Food Insecure	382	2.4
Food Secure	14,508	97.6

PEI: Prince Edward Island, NWT: Northwest Territories.

**Table 4:** Household Food Security Survey Module (Adult Scale). Breakdown of response frequency by questionnaire item for all respondents aged 65+ years, Canadian Community Health Survey (CCHS) 2012.

Survey Items	Positive Responses “Yes” or “Sometimes True” or “Often True” (% of Total Validated Responses)
Worried whether food would run out before getting money to buy more (in last 12 months)	3.0
The food bought didn’t last and there was not enough money to get more (in last 12 months)	2.5
Could not afford to eat balanced meals (in last 12 months)	2.9
*If respondents responded often or sometimes to any of the above questions, they were subsequently asked the following questions:*	
In the past 12 months, did you or other adults in the household ever cut the size of your meals or skip meals	19.7
In the past 12 months, did you ever eat less than you felt you should because there wasn’t enough money to buy food	21.5
In the past 12 months, were you ever hungry because you didn’t eat because you couldn’t afford enough food	9.0
In the past 12 months, did you lose weight because you didn’t have enough money for food	7.2
In the past 12 months, did you or other adults in your household ever not eat for a whole day because you did not have enough money	11.5

**Table 5:** Prevalence of food insecurity among older people aged 65+ years according to selected characteristics (*n* = 14,890).

Subgroup	Any Level of Food Insecurity, % (n)	Prevalence Ratio	χ^2^, *p*-Value
All	2.6 (382/14,890)		
Sex			
Male	2.0 (126/6156)	1.0	11.30 (df = 1), *p* = 0.001
Female	2.9 (256/8734)	1.4	
Age			
65–74 years	3.4 (282/8301)	2.4	51.91 (df = 1), *p* < 0.00005
75+ years	1.5 (100/6589)	1.0	
Racial Background			
White	2.2 (313/14,009)	1.0	103.90 (df = 1), *p* < 0.00005
Visible Minority	7.8 (69/881)	3.5	
Self-Rated Health			
Excellent/Good	1.4 (90/6593)	1.0	68.21 (df = 1), *p* < 0.00005
Fair/Poor	3.5 (292/8297)	2.6	
*Education (Household)*			
Less than Secondary	3.4 (129/3815)	1.5	25.00 (df = 3), *p* < 0.00005
Graduated Secondary	2.1 (48/2277)	0.9	
Other Post-Secondary	4.7 (22/470)	2.1	
Graduated Post-Secondary	2.2 (183/8145)	1.0	
Total Income (Household)			
>$20,000	7.9 (209/2654)	54.1	431.36 (df = 3), *p* <0.00005
$20,000–$39,900	2.7 (148/5481)	18.5	
$40,000–$59,900	0.6 (20/3322)	4.1	
$60,000+	0.1 (5/3433)	1.0	
Marital Status			
Married, Common-Law	1.4 (111/7859)	1.0	88.53 (df = 1), *p* < 0.00005
Other	3.9 (271/6760)	2.8	
Living Arrangement			
Living with spouse	1.3(98/7334)	1.0	89.34 (df = 2), *p* < 0.00005
Living alone	3.8 (17/590)	2.2	
Living with others	2.9 (267/6966)	2.9	
Home Ownership			
Owned	1.5 (168/11,435)	1.0	236.96 (df = 1), *p* < 0.00005
Rented	6.2 (214/3455)	4.2	
Province			
Newfoundland	2.0 (8/403)	1.0	51.97 (df = 10), *p* < 0.00005
PEI	3.8 (8/210)	1.9	
Nova Scotia	3.0 (18/607)	1.4	
New Brunswick	4.1 (25/614)	2.0	
Quebec	2.2 (56/2601)	1.1	
Ontario	2.2 (121/5417)	1.1	
Manitoba	2.8 (24/868)	1.4	
Saskatchewan	2.9 (27/939)	1.4	
Alberta	2.0 (23/1123)	1.0	
British Columbia	2.9 (58/1983)	1.4	
Yukon/NWT/Nunavut	11.2 (14/125)	5.5	

**Table 6:** Base model with four predictors—Age, Sex, Race, Income. The dependent variable is food insecure.

Base Model			
Predictors	Odds Ratio	CI (95%)	*p*-Value
Age (65–74 years ref)	0.314	0.205–0.481	*p* < 0.001
Sex (Male ref)	1.052	0.719–1.539	*p* = 0.793
Race (White ref)	2.793	1.680–4.641	*p* <0.001
Household Income ($60,000+ ref)			
$40,000–$60,000	2.646	0.690–10.142	*p* = 0.156
$20,000–$39,999	16.597	4.759–57.882	*p* < 0.001
<$20,000	62.458	17.816–218.958	*p* < 0.001

**Table 7:** Final multivariable model based on model selection process. The dependent variable is food insecure.

Final Model			
Predictors (Referent Group)	Odds Ratio	CI (95%)	*p*-Value
Age (65–74 years ref)	0.322	0.212–0.419	*p* < 0.001
Sex (Male ref)	1.111	0.762–1.621	*p* = 0.584
Race (White ref)	2.429	1.438–4.102	*p* < 0.001
Household Income ($60,000+ ref)			
$40,000–$60,000	2.527	0.656–9.728	*p* = 0.178
$20,000–$39,999	14.238	4.013–50.522	*p* < 0.001
<$20,000	46.146	12.523–170.041	*p* < 0.001
Home Ownership (Own ref)	2.293	1.561–3.369	*p* = 0.075
Self-Perceived Health (excellent, very good, good ref)	1.449	0.964–2.177	*p* = 0.094
Education (post-graduate ref)			
Other post-secondary	1.363	0.735–2.526	*p* = 0.326
Secondary graduate	0.805	0.450–1.438	*p* = 0.463
<Secondary	0.553	0.364–0.840	*p* < 0.005
Province (Central ref)			
Western(BC, AB, SK, MN)	1.752	1.160–2.645	*p* < 0.001
Atlantic (NFLD, PEI, NS, NB)	1.641	1.046–2.576	*p* < 0.05
Northern (Yukon/NWT/Nunavut)	5.189	2.329–11.562	*p* < 0.001

Newfoundland (NFLD), Prince Edward Island (PEI), Nova Scotia (NS), New Brunswick (NB), Saskatchewan (SK), British Columbia (BC), Alberta (AB), Manitoba (MN).
